# A185 SUSTAINED SYMPTOM CONTROL WITH MIRIKIZUMAB IN PATIENTS WITH MODERATELY TO SEVERELY ACTIVE ULCERATIVE COLITIS IN THE LUCENT-2 MAINTENANCE TRIAL

**DOI:** 10.1093/jcag/gwac036.185

**Published:** 2023-03-07

**Authors:** A Dignass, S Danese, K Matsuoka, M Ferrante, M Long, I Redondo, T H Gibble, R Moses, X Li, N Morris, C Milch, M Abreu, J Jones

**Affiliations:** 1 Agaplesion Markus Krankenhaus, Medizinische Klinik I, Frankfurt, Germany; 2 Vita-Salute San Raffaele University - IRCCS San Raffaele Scientific Institute, Milan, Italy; 3 Gastroenterology and Hepatology, Tokyo Medical and Dental University, Tokyo, Japan; 4 Department of Gastroenterology and Hepatology, University Hospitals Leuven, Leuven, Belgium; 5 University of North Carolina at Chapel Hill, Chapel Hill, United States; 6 Produtos Farmacêuticos, Lda., Eli Lilly Portugal, Lisbon, Portugal; 7 Eli Lilly and Company, Indianapolis; 8 Miller School of Medicine, Crohn's and Colitis Center, University of Miami, Miami, United States; 9 Department of Medicine, Department of Community Health and Epidemiology, Dalhousie University, Halifax, Canada

## Abstract

**Background:**

Mirikizumab (miri) improved symptom control in a Phase 3, multicenter, randomized, double-blind, parallel, placebo-controlled induction study at Week (W)12, in patients (pts) with moderately-to-severely active ulcerative colitis (UC; LUCENT-1).

**Purpose:**

This analysis assessed sustained symptom control during the maintenance phase through W40 (W52 of continuous therapy), among pts who were induced into clinical response with miri.

**Method:**

During the 40W maintenance study (LUCENT-2), pts (N=544) who achieved clinical response to miri 300mg Q4W by W12 of induction, were re-randomized 2:1 to subcutaneous (SC) miri 200mg (n=365) or PBO Q4W (n=179). We evaluated sustained control of stool frequency (SF), rectal bleeding (RB), bowel movement urgency (BU) and abdominal pain (AP). The proportion of pts achieving SF Remission (defined as SF=0, or SF=1 with a ≥1-point decrease from induction baseline [BL]), RB Remission (RB=0), Symptomatic Remission (both SF and RB Remission), Stable Maintenance of Symptomatic Remission (defined as pts in Symptomatic Remission for at least 7 out of 9 visits from W4 to W36 and also at Week 40 among pts in Symptomatic Remission and Clinical Response at the end of LUCENT-1), and AP Improvement (Numeric Rating Scale [NRS] pain score ≥30% improvement from BL in pts with baseline AP NRS ≥3) were assessed. BU NRS change from baseline, and the proportion of pts achieving BU Remission (NRS 0 or 1 in pts with BU NRS ≥3 at baseline) were evaluated.

**Result(s):**

A greater proportion of miri-treated pts achieved SF Remission, RB Remission and Symptomatic Remission compared to PBO at W40 (Table), with significant differences observed from W8 of LUCENT-2 (p=0.042; p=0.004; p=0.036, respectively) and maintained through W40. Miri-treated pts had a significantly higher percentage of Stable Maintenance of Symptomatic Remission (p<0.001). Pts in the miri-treatment group had a significantly greater mean reduction in BU NRS change from induction BL starting at W12 (p=0.034) onwards compared to PBO (Table). Pts assigned to miri accrued an additional 13.6 percentage-point benefit in BU Remission during the first 8W of maintenance therapy and achieved a significant greater improvement at W40 compared to PBO (p<0.001, Table). Similarly, AP was significantly improved for the miri-treated group starting at W16 (p=0.034) onwards compared to PBO.

**Image:**

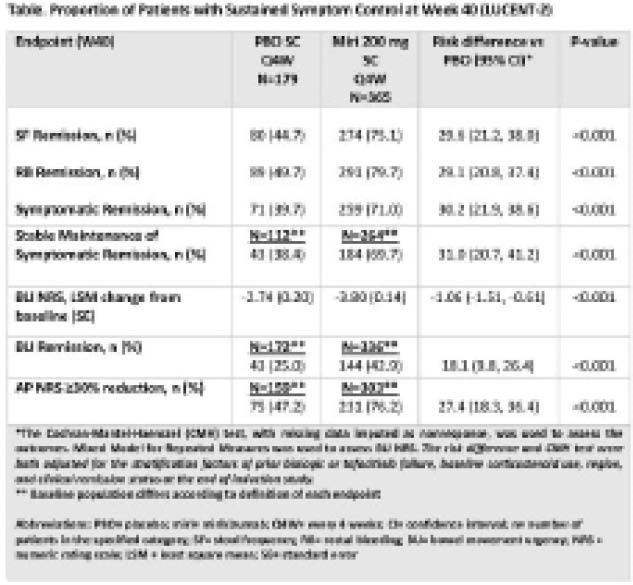

**Conclusion(s):**

Miri provides sustained control of UC symptoms including BU, RB, and SF compared to PBO in pts with moderately to severely active UC.

**Please acknowledge all funding agencies by checking the applicable boxes below:**

Other

**Please indicate your source of funding;:**

Eli Lilly and Company

**Disclosure of Interest:**

A. Dignass Consultant of: AbbVie, Abivax, Amgen, Arena Pharmaceuticals, Bristol Myers Squibb (Celgene), Celltrion, Dr. Falk Pharma, Eli Lilly and Company, Ferring Pharmaceuticals, Fresenius Kabi, Galapagos, Gilead Sciences, Janssen, Merck Sharp & Dohme, Novartis, Pfizer, Pharmacosmos, Roche, Sandoz/Hexal, Takeda, Tillotts Pharma AG, and Vifor Pharma; has received lecture fees or honoraria from: AbbVie, Amgen, Bristol Myers Squibb, Dr. Falk Pharma, Ferring Pharmaceuticals, Galapagos, High5Md, Janssen, Materia, Merck Sharp & Dohme, Pfizer, Sandoz, Takeda, Tillotts Pharma AG, and Vifor Pharma, S. Danese Consultant of: AbbVie, Alimentiv, Allergan, Amgen, AstraZeneca, Athos Therapeutics, Biogen, Boehringer Ingelheim, Bristol Myers Squibb, Celgene, Celltrion, Dr. Falk Pharma, Eli Lilly and Company, Enthera, Ferring Pharmaceuticals, Gilead Sciences, Hospira, Inotrem, Janssen, Johnson & Johnson, Merck Sharp & Dohme, Mundipharma, Mylan, Pfizer, Roche, Sandoz Sublimity, Takeda, TiGenix, UCB Pharma, and Vifor Pharma, Speakers bureau of: AbbVie, Amgen, Ferring Pharmaceuticals, Gilead Sciences, Janssen, Mylan, Pfizer, and Takeda, K. Matsuoka Grant / Research support from: AbbVie, EA Pharma, JIMRO, Kissei Pharmaceutical, Kyowa Kyorin, Mitsubishi Tanabe, Mochida Pharmaceutical, and Zeria Pharmaceutical Nippon; lecture fees from: AbbVie, EA Pharma, JIMRO, Kissei Pharmaceutical, Kyowa Kyorin, Mitsubishi Tanabe, Mochida Pharmaceutical, Takeda, and Zeria Pharmaceutical Nippon, M. Ferrante Grant / Research support from: AbbVie, Amgen, Biogen, Janssen Cilag, Pfizer, Takeda, and Viatris, Consultant of: AbbVie, Boehringer Ingelheim, Celltrion, Eli Lilly and Company, Janssen Cilag, Medtronic, Merck Sharp & Dohme, Pfizer, Regeneron, Sandoz, Takeda, and Thermo Fisher Scientific, Speakers bureau of: AbbVie, Amgen, Biogen, Boehringer Ingelheim, Celltrion, Dr. Falk Pharma, Eli Lilly and Company, Ferring Pharmaceuticals, Janssen, Lamepro, Medtronic, Merck Sharp & Dohme, Mylan, Pfizer, Samsung Bioepis, Sandoz, Takeda, and Thermo Fisher Scientific, M. Long Consultant of: AbbVie, Bristol Myers Squibb, Calibr, Eli Lilly and Company, Genentech, Janssen, Pfizer, Prometheus Biosciences, Roche, Takeda, TARGET PharmaSolutions, and Theravance Biopharma, I. Redondo Employee of: Eli Lilly and Company, T. Gibble Employee of: Eli Lilly and Company, R. Moses Employee of: Eli Lilly and Company, X. Li Employee of: Eli Lilly and Company, N. Morris Employee of: Eli Lilly and Company, C. Milch Employee of: Former employee, was employed at Eli Lilly and Company at the time of study, M. Abreu Grant / Research support from: Pfizer, Prometheus Biosciences, and Takeda, Consultant of: AbbVie, Arena Pharmaceuticals, Bristol Myers Squibb, Eli Lilly and Company, Gilead Sciences, Janssen, Microba Life Sciences, Prometheus Biosciences, UCB Pharma, and WebMD, Speakers bureau of: Alimentiv, Intellisphere LLC (HCP Live Institutional Perspectives in GI), Janssen, Prime CME, and Takeda, J. Jones: None Declared

